# A century of genetic variation inferred from a persistent soil‐stored seed bank

**DOI:** 10.1111/eva.12675

**Published:** 2018-07-29

**Authors:** Jennifer L. Summers, Brittany Bernik, Colin J. Saunders, Jason S. McLachlan, Michael J. Blum

**Affiliations:** ^1^ Department of Ecology and Evolutionary Biology Tulane University New Orleans Louisiana; ^2^ Southeast Environmental Research Center Florida International University Miami Florida; ^3^ Department of Biological Sciences University of Notre Dame Notre Dame Indiana

**Keywords:** climate change, coastal marsh, resurrection ecology, *Schoenoplectus americanus*, *Scirpus olneyi*, sedge

## Abstract

Stratigraphic accretion of dormant propagules in soil can result in natural archives useful for studying ecological and evolutionary responses to environmental change. Few attempts have been made, however, to use soil‐stored seed banks as natural archives, in part because of concerns over nonrandom attrition and mixed stratification. Here, we examine the persistent seed bank of *Schoenoplectus americanus*, a foundational brackish marsh sedge, to determine whether it can serve as a resource for reconstructing historical records of demographic and population genetic variation. After assembling profiles of the seed bank from radionuclide‐dated soil cores, we germinated seeds to “resurrect” cohorts spanning the 20th century. Using microsatellite markers, we assessed genetic diversity and differentiation among depth cohorts, drawing comparisons to extant plants at the study site and in nearby and more distant marshes. We found that seed density peaked at intermediate soil depths. We also detected genotypic differences among cohorts as well as between cohorts and extant plants. Genetic diversity did not decline with depth, indicating that the observed pattern of differentiation is not due to attrition. Patterns of differentiation within and among extant marshes also suggest that local populations persist as aggregates of small clones, likely reflecting repeated seedling recruitment and low immigration from admixed regional gene pools. These findings indicate that persistent and stratified soil‐stored seed banks merit further consideration as resources for reconstructing decadal‐ to century‐long records that can lend insight into the tempo and nature of ecological and evolutionary processes that shape populations over time.

## 1 INTRODUCTION

Stratigraphic accretion of dormant propagules in soil can result in natural archives useful for studying ecological and evolutionary responses to environmental change (Hansen, 2012). Ephippia (i.e., resting stage eggs) of freshwater zooplankton recovered from lake sediments, for example, have been leveraged to reconstruct decadal‐ to century‐long records of response to environmental degradation including acidification, eutrophication, heavy metal contamination, and warming (e.g., Brede et al., 2009; Brendonck & De Meester, 2003; De Meester, Van Doorslaer, Geerts, Orsini, & Stoks, 2011; Derry, Arnott, & Boag, 2010; Hairston et al., 1999; Kerfoot, Robbins, & Weider, 1999; Limburg & Weider, 2002; Mergeay, Vanoverbeke, Verschuren, & Meester, 2007; Pollard, Colbourne, & Keller, 2003; Weider, Lampert, Wessels, Colbourne, & Limburg, 1997). Like resting eggs in lake sediments, seed banks have proven to be useful natural archives. Seeds recovered from shallow soils and aerial banks (i.e., seeds retained on parent trees) can serve as resources for understanding the magnitude and structure of genetic variation across successive life history stages (Ayre, O'Brien, Ottewell, & Whelan, 2010; Barrett, He, Lamont, & Krauss, 2005; Cabin, Mitchell, & Marshall, 1998; Hock, Szövényi, Schneller, Tóth, & Urmi, 2008; Zipperle, Coyer, Reise, Stam, & Olsen, 2009). Seeds have been revived from stored collections to assess microevolutionary responses to recent climate‐related environmental change (Franks, 2011; Franks, Sim, & Weis, 2007; Franks & Weis, 2008; Franks & Weis, 2009; Sultan, Horgan‐Kobelski, Nichols, Riggs, & Waples, 2013). Seeds in time‐stratified sediments also are often used for paleoecological reconstruction of plant community composition over time (e.g., Jarrell, Kolker, Campbell, & Blum, 2016; Törnqvist et al., 2004). Few attempts have been made, however, to reconstruct historical records of genetic variation to infer ecological and evolutionary responses of plants to environmental change from time‐stratified soil‐stored seed banks (Bennington, McGraw, & Vavrek, 1991; Gugerli, Parducci, & Petit, 2005; McGraw, 1993; Morris, Baucom, & Cruzan, 2002; Vavrek, McGraw, & Bennington, 1991).

Biased representation and poor stratification are two well‐recognized concerns that have deterred use of soil‐stored seed banks for reconstructing records of genetic variation and other aspects of organismal evolution (Brendonck & De Meester, 2003; Franks & Weis, 2008; Weis, 2018). Bias can arise because, for many plants, only a fraction of seeds that fall to the ground enter the seed bank (Templeton & Levin, 1979). Nonrandom attrition of buried seeds or selection acting on traits associated with germination can further bias the composition of a seed bank over time (Weis, 2018). Mixing or weak stratification of soil layers also can confound relative and absolute aging of buried propagules (Brendonck & De Meester, 2003; Franks & Weis, 2008; Hairston & Kearns, 2002). Steps can be taken, however, to mitigate both concerns. For example, targeting a species with prolific seed production can reduce the likelihood of biased representation and false signatures of selection (Brendonck & De Meester, 2003; Weider et al., 1997). In addition, seeds from distinct depth ranges can be treated as age‐relative “cohorts” (Morris et al., 2002) and, like resting stage eggs, seeds can be precisely dated when recovered from depositional environments, such as freshwater lakes and coastal wetlands, with highly stratified sediments (Bennington et al., 1991; Brendonck & De Meester, 2003; Jarrell et al., 2016; Törnqvist et al., 2004; Vavrek et al., 1991).

Prior use of the soil‐stored seed bank of the foundational coastal marsh sedge *Schoenoplectus americanus* (Pers.) Volkart ex Schinz & R. Keller (Cyperaceae) for studying paleoecological responses to environmental change (e.g., Jarrell et al., 2016; Saunders, 2003; Törnqvist et al., 2004) indicates that it also could be a valuable resource for reconstructing historical records of genetic variation. Formerly known as *Scirpus olneyi* (and commonly known as chairmaker's bulrush and Olney's bulrush), *S. americanus* has been the focus of more than three decades of research on coastal marsh responses to climate change (e.g., Arp, Drake, Pockman, Curtis, & Whigham, 1993; Blum, McLachlan, Saunders, & Herrick, 2005; Broome, Mendelssohn, & McKee, 1995; Drake, 2014; Langley, McKee, Cahoon, Cherry, & Megonigal, 2009; Langley & Megonigal, 2010; Langley, Mozdzer, Shepard, Hagerty, & Megonigal, 2013; Rasse, Peresta, & Drake, 2005; Saunders, Megonigal, & Reynolds, 2006). Annual production of a prolific number of seeds with exceptionally durable coats (Miller, Smeins, Webb, & Longnecker, 1997; Sherfy & Kirkpatrick, 1999) can result in highly stratified seed banks that persist for decades to millennia (Brush, 2001; Jarrell et al., 2016; Saunders, 2003; Törnqvist et al., 2004). Profiles of *S. americanus* seed banks have been used to infer shifts in relative abundance over time, as *S. americanus* seed production correlates with peak season aboveground biomass (Jarrell et al., 2016; Saunders, 2003). Seed bank profiles of *S. americanus* also have served as a resource for paleoecological reconstruction of marsh responses to sea level rise (Jarrell et al., 2016; Saunders, 2003; Törnqvist et al., 2004) because the contribution of *S. americanus* primary production to soil organic matter accumulation is mediated by estuarine salinity (Choi, Wang, Hsieh, & Robinson, 2001; Rasse et al., 2005; Ross & Chabreck, 1972). Depending on the condition of buried seeds, it also might be possible to characterize genetic variation over time to draw further inferences about the tempo and nature of *S. americanus* responses to environmental change.

In this study, we evaluated the extent to which soil‐stored seed banks of *S. americanus* can serve as natural archives for reconstructing historical records of demographic and genetic variation. We first assessed whether sediments exhibited a time‐stratified structure characteristic of recurring deposition and accumulation. We then assessed whether seed densities steadily declined with soil depths or exhibited variation consistent with shifts in the abundance of *S. americanus* through time (Jarrell et al., 2016). We also assessed whether genetic diversity declined with increasing soil depth, which can result from attrition or differences in germination bias (Orsini et al., 2016). In addition, we assessed whether estimates of genetic structure and pairwise measures of genetic distance varied erratically with increasing soil depth, which can also result from nonrandom attrition and bias. We did so by first reconstructing the stratigraphy of buried seeds from ^210^Pb and ^137^Cs dated soil cores. We then germinated seeds to “resurrect” and genotype cohorts spanning the 20th century. Using a suite of microsatellite markers, we inferred patterns of genetic diversity and differentiation among “resurrected” cohorts, drawing comparisons to extant plants at the coring site as well as in nearby and more distant marshes across the Atlantic and Gulf coasts. In addition to offering perspective on the potential importance of nonrandom bias, this approach enabled us to infer whether patterns of temporal variation reflect immigration or local population differentiation (Holt, 1990). It also enabled us to bypass concerns about DNA contamination of buried seeds (Anderson‐Carpenter et al., 2011; Gugerli et al., 2005) and assess whether soil‐stored seed banks can serve as resources for assembling experimental populations to study adaptive evolution to contemporary environmental change (Franks et al., 2007).

## 2 METHODS

### 2.1 Soil excavation site, seed recovery and estimation of accretion rates

We excavated sediment cores from Kirkpatrick Marsh (Table 1), which is the site of the Global Change Research Wetland (GCReW) operated by the Smithsonian Environmental Research Center (Arp et al., 1993; Broome et al., 1995; Rasse et al., 2005). The GCReW has supported several studies that span 30+ years of investigation (e.g., Curtis, Drake, & Whigham, 1989; Lu et al., 2016) of ecosystem‐level responses to elevated CO_2_ (Drake, 2014), nitrogen (Langley & Megonigal, 2010), invasive species (Caplan, Hager, Megonigal, & Mozdzer, 2015), and warming (Megonigal et al., 2016). As a dominant species of the GCReW plant community, *S. americanus* has featured prominently in much of this work. Kirkpatrick Marsh borders the Rhode River, a subestuary of Chesapeake Bay near Edgewater, Maryland (38º 51′N, 76º 32′W). Elevation of the marsh is 40–60 cm above mean low water, with 20% of high tides flooding the site (Jordan, Pierce, & Correll, 1986). Soil salinity ranges from 2 ppt to 18 ppt during the growing season (May to September), where interannual variation in growing season salinity is inversely correlated with rainfall (Saunders, 2003).

**Table 1:** Recovery and germination of seeds from Kirkpatrick Marsh soil cores

					Core 2004‐A		Core 2004‐B		Monolith( )assay #1		Monolith assay #2	
Soil Layer (cm)	Soil Date (cal year)	*N*	*N*_G_	%_G_	*N*	*N*_G_	*N*	*N*_G_	*N*	*N*_G_	*N*	*N*_G_
0–2	2002 ± 0.1	8	2	25	8	2						
2–4	1998 ± 0.4	165	46	28	4	1			65	6	96	39
4–6	1993 ± 0.6	3	1	33	3	1						
6–8	1990 ± 1.3	7	3	43	7	3						
8–10	1984 ± 1.2	257	60	23	10	1	96	24	55	25	96	10
10–12	1976 ± 1.2	87	34	39	87	34						
12–14	1963 ± 3.0	187	41	22	187	41						
14–16	1947 ± 4.2	337	8	2	90	2	96	0	55	3	96	3
16–18	1933 ± 7.2	92	2	2	92	2						
18–20	1918 ± 15.6	120	0	0	120							
20–22	1908 ± 25.0	376	11	3	192	1	96	1	60	8	28	1
22–24	1900 ± 32.8	479	1	0	250		192	0	25	1	12	0
24–26	1891 ± 43.8	52	0	0	52							
26–28	1884 ± 54.7	1	0	0	1							
28–30	1875 ± 92.8	1	0	0	1							
34–36		1	0	0	1							
36–38		18	0	0	18							
38–40		5	0	0	5							
42–44		1	0	0	1							
54–56		1	0	0	1							
56–58		5	0	0	5							
60–62		1	0	0	1							

**Figure 1:**
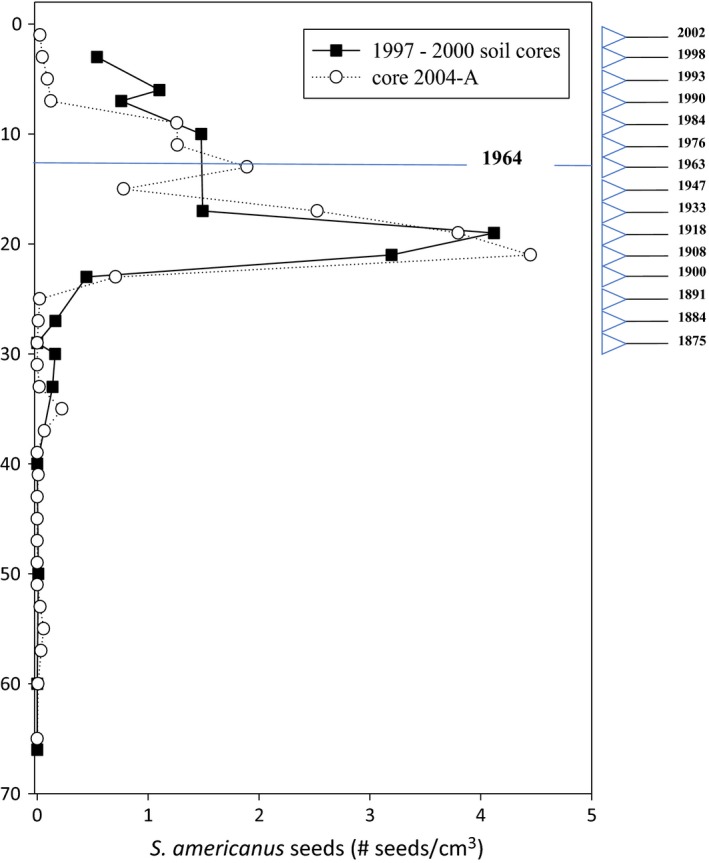
Profile of the *Schoenoplectus americanus* seed bank in Kirkpatrick Marsh. Relative abundance of *S. americanus* seeds recovered from a series of soil cores taken in Kirkpatrick Marsh. Estimated dates of soil depths from ^137^Cs (blue line = max concentration) and ^210^Pb (right outset) according to a Constant‐Flux variable sedimentation rate model

We reconstructed soil stratigraphy and seed bank profiles from a set of soil cores taken in Kirkpatrick Marsh. As described by Saunders (2003) and Saunders et al. (2006), a series of 70‐cm‐deep piston cores (5.1 cm diameter) were excavated between 1997 and 2000 at four‐month intervals for a study quantifying depth profiles of C_3_ and C_4_ belowground biomass in eleven 1.5‐m^2^ plots in the marsh. Soil core samples from a 1.5‐m^2^ plot with equal amounts of C_3_ (*S. americanus*) and C_4_ (e.g., *Spartina patens*,* Distichlis spicata*) aboveground biomass were used to quantify a vertical profile of *S. americanus* seeds (Table 1, Figure 1). In October 2002, we removed a supplemental 30 cm diameter × 35 cm deep core (hereafter referred to as a “soil monolith”) to recover additional *S. americanus* seeds for germination assays. The soil monolith was taken adjacent to the mixed C_3_‐C_4_ study plot (Plot #15; Table 2) where the 1997–2000 cores were taken to reconstruct the seed bank profile. In addition, in February 2004, we removed two more piston cores (15.2 cm diameter, 30 cm apart) 2 m from where the soil monolith was taken to further quantify the vertical profile of *S. americanus* seeds (Figure 1), to recover more seeds for germination assays (Table 1), and to estimate accretion rates.

**Table 2:** Genetic variation of “resurrected” and extant *Schoenoplectus americanus* from Kirkpatrick Marsh

								Clones excluded			Clones included		
Site No.	Location	State	Latitude (N)	Longitude (W)	*N*	*G*	*N*_e_	*R*	*H*_e_	*S*	*H*_e_	*S*	*A*_R_
Kirkpatrick marsh													
1	Kirkpatrick marsh, plot 1 (Sp, A)	MD	38°52′26.62″	76°32′55.64″	6	6		1.00	0.27	0.47	0.42	0.60	1.42
2	Kirkpatrick marsh, plot 2 (Sp, A)	MD	38°52′27.55″	76°32′56.77″	3	3		1.00	0.30	0.38	0.30	0.38	1.30
3	Kirkpatrick marsh, plot 3 (Sp, E)	MD	38°52′27.18″	76°32′59.68″	5	4		0.80	0.24	0.32	0.39	0.44	1.39
4	Kirkpatrick marsh, plot 4 (Sp, A)	MD	38°52′26.97″	76°32′58.61″	1	1		1.00	0.60	0.42	1.00	0.60	0.42
5	Kirkpatrick marsh, plot 6 (Sc, A)	MD	38°52′25.63″	76°32′57.10″	3	3		1.00	0.38	0.50	0.38	0.50	1.38
6	Kirkpatrick marsh, plot 7 (Sc, A)	MD	38°52′25.55″	76°32′57.10″	3	1		0.33	0.27	0.32	0.45	0.32	1.45
7	Kirkpatrick marsh, plot 8 (Sc, E)	MD	38°52′26.44″	76°32′57.55″	1	1		1.00	0.45	0.32	0.45	0.32	1.45
8	Kirkpatrick marsh, plot 10 (Sc, A)	MD	38°52′26.44″	76°32′57.40″	2	2		1.00	0.41	0.49	0.41	0.49	1.41
9	Kirkpatrick marsh, plot 11 (Sc, E)	MD	38°52′26.28″	76°32′57.14″	4	4		1.00	0.41	0.55	0.41	0.55	1.41
10	Kirkpatrick marsh, plot 12 (Sc, A)	MD	38°52′26.75″	76°32′57.37″	4	1		0.25	0.26	0.41	0.48	0.65	1.48
11	Kirkpatrick marsh, plot 13 (Sc, E)	MD	38°52′26.59″	76°32′57.08″	5	2		0.40	0.26	0.38	0.31	0.44	1.31
12	Kirkpatrick marsh, plot 14 (Sc, E)	MD	38°52′26.76″	76°32′57.12″	5	1		0.20	0.30	0.39	0.39	0.55	1.39
13	Kirkpatrick marsh, plot 15 (Sc, E)	MD	38°52′26.89″	76°32′56.90″	5	2		0.40	0.21	0.32	0.52	0.59	1.52
14	Kirkpatrick marsh, plot 17 (Sc, A)	MD	38°52′27.09″	76°32′56.80″	3	2		0.67	0.28	0.29	0.41	0.45	1.41
15	Kirkpatrick marsh, plot 18 (Mx, A)	MD	38°52′29.03″	76°32′59.68″	3	3		1.00	0.24	0.28	0.24	0.28	1.24
16	Kirkpatrick marsh, plot 19 (Mx, A)	MD	38°52′28.91″	76°32′59.54″	3	1		0.33	0.22	0.25	0.36	0.25	1.36
17	Kirkpatrick marsh, plot 20 (Mx, E)	MD	38°52′27.76″	76°33′0.27″	8	5		0.63	0.10	0.13	0.23	0.32	1.23
18	Kirkpatrick marsh, plot 21 (Mx, A)	MD	38°52′27.74″	76°33′0.36″	3	1		0.33	0.12	0.13	0.33	0.12	0.13
19	Kirkpatrick marsh, plot 22 (Mx, A)	MD	38°52′27.85″	76°33′0.10″	5	4		0.80	0.09	0.13	0.09	0.13	1.09
20	Kirkpatrick marsh, plot 23 (Mx, E)	MD	38°52′28.00″	76°32′59.80″	5	1		0.20	0.05	0.06	0.20	0.05	0.06
21	Kirkpatrick marsh, plot 24 (Mx, A)	MD	38°52′28.08″	76°32′59.57″	5	1		0.20	0.05	0.06	0.20	0.05	0.06
22	Kirkpatrick marsh, plot 25 (Mx, E)	MD	38°52′27.51″	76°32′59.70″	3	1		0.33	0.27	0.32	0.33	0.27	0.32
23	Kirkpatrick marsh, plot 26 (Mx, A)	MD	38°52′27.45″	76°32′59.53″	4	1		0.25	0.27	0.36	0.27	0.19	1.27
24	Kirkpatrick marsh, plot 27 (Mx, E)	MD	38°52′27.72″	76°32′59.21″	5	5		1.00	0.17	0.22	0.47	0.49	1.47
25	Kirkpatrick marsh, plot 28 (Mx, A)	MD	38°52′27.46″	76°32′59.11″	5	1		0.20	0.15	0.19	0.20	0.15	0.19
26	Kirkpatrick marsh, plot 29 (Mx, E)	MD	38°52′27.27″	76°32′58.77″	5	4		0.80	0.34	0.42	0.41	0.43	1.41
27	Kirkpatrick marsh, plot 30 (Mx, A)	MD	38°52′27.21″	76°32′58.50″	5	1		0.20	0.35	0.43	0.20	0.35	0.43
				Total	109	70		0.60	0.26	0.32	0.37	0.42	1.37
Kirkpatrick marsh, depth cohorts													
	Kirkpatrick marsh, 2–4 cm (1998)	MD	38°52′26.63″	76°32′53.16″	12	12	1346.9	1.00	0.37	0.64	0.37	0.64	1.37
	Kirkpatrick marsh, 8–10 cm (1984)	MD	38°52′26.63″	76°32′53.16″	5	5	8.9	1.00	0.44	0.70	0.44	0.70	1.44
	Kirkpatrick marsh, 12–14 cm (1963)	MD	38°52′26.63″	76°32′53.16″	40	40	57.4	1.00	0.42	0.82	0.42	0.82	1.42
	Kirkpatrick marsh, 14–16 cm (1947)	MD	38°52′26.63″	76°32′53.16″	9	9	25.2	1.00	0.43	0.69	0.43	0.69	1.43
	Kirkpatrick marsh, 20–24 cm (1900–1908)	MD	38°52′26.63″	76°32′53.16″	9	9	2.4	1.00	0.45	0.76	0.45	0.76	1.45
				Total	75	75		1.00	0.42	0.72	0.42	0.72	1.42
				Overall total	184	145							

Plot numbers correspond to those given in Figure 3, where Sp = *Spartina patens*‐dominated plots; Sc = *Schoenoplectus americanus*‐dominated plots; Mx = mixed species plots; and A = ambient CO_2_; E = elevated CO_2_. Sample size is given as (*N*); *G* = number of unique multilocus genotypes; *R* = genotypic richness; *H*_e_ = expected heterozygosity; *S* = Shannon diversity index values; *A*_R_ = rarified allelic richness; and *N*_e_ = effective population size. Diversity measures were calculated with and without putative clones. Putative clones were excluded from *N*_e_ calculations.

Following removal, all sampled soil was transported to Duke University for processing and analysis. The 2002 soil monolith was sliced into 2 cm increments perpendicular to the vertical axis for recovery and germination of *S. americanus* seed cohorts (Table 1). The first 2004 piston core (“core 2004‐A,” 65 cm deep) was also cut into 2 cm layers, with one half of each layer dry‐sieved over a 2 mm mesh (to remove large roots and rhizomes) in preparation for radionuclide analysis of ^210^Pb and ^137^Cs (Saunders, 2003). Soil dates from ^210^Pb radionuclide data were estimated according to the constant rate of supply model (Appleby & Oldfield, 1978) to allow for variable accretion over time, as accretion rates in Chesapeake Bay marshes have fluctuated over the last 200 years (Kearney, 1996; Kearney, Stevenson, & Ward, 1994). Variability in soil dates was calculated by first‐order error analysis of counting uncertainty (Binford, 1990). The depth of peak ^137^Cs activity was used as an independent marker of the depth corresponding to 1964, the year when ^137^Cs reached peak concentrations in the atmosphere. The remaining soil from core 2004‐A was used to recover additional seeds for germination and for reconstructing the seed bank profile (Table 1, Figure 1). The second piston core (“core 2004‐B”; 40 cm deep) was used to recover additional seeds from soil horizons deeper than 8 cm for germination assays.

### 2.2 Seed germination and tissue sampling of “resurrected” cohorts

We conducted two germination assays to assess seed viability as well as to “resurrect” and genotype plants from buried seeds (e.g., Härnström, Ellegaard, Andersen, & Godhe, 2011; Kerfoot, Budd, Eadie, Vanderploeg, & Agy, 2004; Kerfoot & Weider, 2004; Zipperle et al., 2009). We conducted the first germination assay from February to March 2003 to evaluate the viability of seeds recovered from the 2002 soil monolith. Seeds from the 2–4, 8–10, 14–16, 20–22, and 22–24 cm layers (Table 1) of the monolith were planted in a 1:2 mixture of sterile sand and Ferry & Morse Seed Starter Mix ^®^ (Ferry & Morse, Fulton, KY, USA). We filled 32 pots with the mixture and arrayed the pots in a rectangular grid within a 6 cm deep tray (24 × 48 cm^2^). The tray was filled with water, and water levels were kept at approximately 1 cm below the soil surface. Seeds from each of the five soil layers were randomly assigned to 2–4 pots per layer with 10–30 seeds placed in each pot. The tray was placed in a growth cabinet with a 15 hr light:9 hr dark photoperiod and 30^°^C constant temperature (due to a mechanical problem, the photoperiod during the first 6 days was 24 hr light:0 hr dark). The number of germinating seeds was recorded daily for the first 7 days and again at 10, 12, 14, and 19 days after planting. The second germination assay was conducted from May to July 2004 involving (a) 328 additional seeds recovered from depths 2–4, 8–10, 14–16, 20–22, and 22–24 cm of the 2002 soil monolith; (b) 1,136 seeds recovered from all depths (0–64 cm) of core 2004‐A; and (c) 480 seeds recovered from depths 8–10, 14–16, 20–22, and 22–24 cm of core 2004‐B (Table 1). All seeds were planted in separate pots, each filled with one part sand and two parts Fafard Professional Formula Seed Starter Potting Mix ^®^ (Conrad Fafard, Inc., Agawam, MA, USA). The assay was conducted in a growth room with a 15‐hr light:9‐hr dark photoperiod and 30^°^C constant temperature. Germination success was recorded as in the first assay. Differences in germination among seed cohorts were assessed using analysis of variance (ANOVA) in *Systat* v.13 (SPSS, Chicago, IL, USA). Bonferroni‐corrected post hoc least‐squares means tests were conducted to compare cohorts. Approximately 0.30 g of leaf tissue was taken from each of 75 individual seedlings resulting from the two germination assays for genetic analysis of the 2–4, 8–10, 12–14, 14–16, 20–22, and 22–24 cm depth cohorts (Tables 1 and 2).

### 2.3 Tissue sampling of extant populations

Tissues were collected for genetic analysis of extant individuals in Kirkpatrick Marsh to better understand patterns of temporal genetic variation. In the growing seasons of 2002 and 2003, a total of 109 tissue samples were collected from *S. americanus* in 27 1.5‐m^2^ plots located within a 130 *× *80 m^2^ section of Kirkpatrick marsh (Table 2). A 10 cm long tissue sample was trimmed from one to six green shoots per plot (Table 2). As *S*. *americanus* can reproduce asexually through vegetative tillering, care was taken to sample evenly across each plot to minimize repeated sampling of the same genet. The relative location of each sample was noted according to the UTM coordinates of the plot, which were spaced ≥2.5 m apart.

The majority of the plots were established in 1987 to study ecological and physiological responses of *S. americanus* and co‐occurring C_4_ species to elevated atmospheric CO_2_ (Arp et al., 1993). Accordingly, these plots differ in CO_2_ exposure regime (Table 2). The remaining plots were established in 1997 for the study of marsh biogeochemistry (Saunders, 2003; Saunders et al., 2006). The vegetative composition of the plots ranged from monospecific stands of *S. americanus,* to mixed communities where *S. americanus* co‐occurs with *S. patens* and other C_4_ plant species, to stands dominated by *S. patens* (Arp et al., 1993; Saunders, 2003; Table 2).

An additional 111 tissues samples were collected from *S. americanus* in nine other marshes across Chesapeake Bay during the 2003 growing season (Supporting Information Table S1). From nine to 19 samples were collected from each location (Supporting Information Table S1). Between 2002 and 2008, another 138 samples were collected from nine other marshes along the Atlantic coast, and 296 samples were collected from 17 marshes along the Gulf of Mexico coast (Supporting Information Table S1). At each location, complete or nearly complete shoots with seed‐bearing inflorescences were taken from plants spaced ≥3 m apart. The coordinates of individual samples from these marshes were not taken. All tissue samples were stored in coolers with ice packs for transport to long‐term storage in −20^°^C freezers.

### 2.4 Genetic data collection

We genotyped all resurrected and extant specimens at 11 microsatellite loci to examine patterns of temporal and spatial genetic variation (Blum et al., 2005). Genomic DNA was extracted from shoot tissue from all samples using DNeasy plant extraction kits (Qiagen, Inc.). The loci SCAM.4, SCAM.5, SCAM.7, SCAM.8, SCAM.11, SCAM.13, SCAM.14, and SCAM.16 described in Blum et al. (2005) were used to genotype all individuals. Three additional loci, corresponding to the following primer sets, were also used in this study: SCAM.17 (forward: 5′‐GCTGACGCTTCCGTAAAAC‐3′; reverse: 5′‐TCCGTTGAG TCCTTGCTCT‐3′), SCAM.18 (forward: 5′‐GTTTCCTGCTTGTCTTTCTG‐3′; reverse: 5′‐CACACCTCTTCTTCCTCTCTT‐3′), and SCAM.19 (forward: 5′‐AACTCCAA AGAACAAACCTTC‐3′; reverse: 5′‐GTGGGAAACAGACTGGTAGTAG‐3′). All 11 loci were designed to anneal at 53°C. Following Blum, Knapke, McLachlan, Snider, and Saunders (2010), we implemented a chloroplast DNA PCR‐RFLP assay to confirm species‐level maternal ancestry to assess whether specimens were of hybrid origin (i.e., to differentiate *S. americanus* from *S. pungens* ancestry; Blum et al., 2005, 2010). This confirmed that all 729 tissue samples used for this study exhibited *S. americanus* species‐level cpDNA ancestry and none exhibited evidence of hybridization (Blum et al., 2010).

For each individual and each microsatellite locus, 10–50 ηg of genomic DNA was used as template in 15 μl PCR mixtures that also included 1U of *Taq* polymerase (Invitrogen), 75 μM of each dNTP, 1 pmole of each primer, and 1× PCR buffer (200 mM Tris‐HCl, pH 8.4; 500 mM KCl). The forward primer was fluorescently labeled with HEX, 6‐FAM, or TET for each primer pair. All PCR amplifications were generated with a thermal regime of 35 cycles of 94°C for 45 s, 53°C for 30 s, and 72°C for 90 s, followed by a final extension stage at 72°C for 5 min. The labeled PCR amplicons were sized against a CST ROX 50‐500 standard (BioVentures, Inc.) on an ABI 3100 Genetic Analyzer (Applied Biosystems, Life Technologies) and scored with *Genemarker* software (Softgenetics, Inc.).

### 2.5 Genetic data analysis

#### 2.5.1 Clonality, genetic diversity, and effective population size

We first determined the number of multilocus genotypes (*G*) and the proportion of samples exhibiting a distinct genotype (*R*) for each depth cohort and sample site using the program *GenAlEx* v.6.41 (Table 2, Supporting Information Table S1) (Peakall & Smouse, 2006). We also assessed the probability that shoots with identical genotypes were members of the same clone using the *Pgen* routine in the program *GenClone* v.2.1 (Arnaud‐Haond & Belkhir, 2007; Parks & Werth, 1993). In addition, we calculated the probability of sampling a second occurrence of each genotype given the number of genets sampled using *Psex* (Parks & Werth, 1993), and we used *GenClone* v.2.1 to calculate the clonal subrange (Alberto et al., 2005; Harada, Kawano, & Iwasa, 1997) of extant *S. americanus* in Kirkpatrick Marsh.

We estimated genetic diversity by first calculating expected heterozygosity (*H*_e_) and Shannon diversity (*S*) including all samples (i.e., without discarding clones) per depth cohort and sample site using *Microsatellite Analyzer* (*MSA)* (Dieringer & Schlotterer, 2003). We also calculated *H*_e_, *S*, and rarified values of allelic richness (*A*_R_) excluding putative clones to account for the possibility of repeated sampling of genetically identical specimens. In addition, we estimated effective population size (*N*_e_) for each depth cohort and for sites sampled for extant *S. americanus*, based on Burrow's composite measure of disequilibrium as implemented in the program *LDNe* (Waples & Do, 2008). Unless otherwise noted, all subsequent analyses were carried out with estimates derived from datasets without putative clones.

With depth serving as a proxy for age, we determined whether genetic diversity differed according to age and location using post hoc least‐squares linear regressions in *Fstat* v.2.93 (Goudet, 1995). We assessed whether there were differences among (a) depth cohorts; (b) all depth cohorts versus all extant *S. americanus* sampled in Kirkpatrick Marsh; (c) extant *S. americanus* in Kirkpatrick Marsh first according to community type (i.e., samples from monospecific stands versus mixed communities) and by CO_2_ regime (i.e., “ambient” versus “elevated” plots); (d) extant *S. americanus* from Kirkpatrick Marsh versus other Chesapeake marshes; and (e) extant *S. americanus* from Atlantic versus Gulf coast locations. The significance of the outcome of each test was determined by comparison of the observed value to 10,000 permutations of samples between groups, with α representing the proportion of randomized data sets giving a larger value than the observed value. All comparisons excluded sites with <3 distinct genotypes.

We tested for declines in genetic diversity with increasing depth‐ an expected outcome of attrition and germination bias (Orsini et al., 2016) ‐using a linear regression and a Kolmogorov–Smirnov test with two potential expected outcomes (i.e., declines in diversity and no change in diversity), both of which were implemented in *R v.3.4.0* (R Core Team, 2013). We similarly tested for declines in *N*_e_ with depth. Using the *R* v.3.4.0 core package (R Core Team, 2013), we also examined correlations between estimates of genetic diversity and *N*_e_ with seed density, which has served as a proxy measure for the relative abundance of *S. americanus* over time (Jarrell et al., 2016; Saunders, 2003).

#### 2.5.2 Genetic and genotypic differentiation

We used *GenAlEx* v.6.41 to conduct an analysis of molecular variance (AMOVA) to examine the distribution of genetic variation across depth cohorts. We also performed AMOVAs with samples grouped according to age (i.e., depth cohorts vs. extant *S. americanus* in Kirkpatrick Marsh) and location (i.e., among Chesapeake Bay marshes, Atlantic versus Gulf coast marshes). In addition, we conducted AMOVAs to assess whether genetic variation in extant *S. americanus* reflects CO_2_ exposure regime and community type (respectively) across the sampled plots in Kirkpatrick Marsh.

We assessed patterns of genetic structure according to allele frequency variation using several complementary methods. Using *Genetix* v.4.05 (Belkhir, Borsa, Chikhi, Goudet, & Bonhomme, 1996), we conducted a factorial correspondence analysis (FCA) of genetic variation in depth cohorts and extant *S. americanus* in Kirkpatrick Marsh. *MSA* was used to calculate and bootstrap the variance in the proportion of shared alleles 1,000 times across depth cohorts and a selection of extant populations to construct a UPGMA dendrogram using the “Neighbor” and “Consense” subroutines of *PHYLIP* v3.63 (Bowcock et al., 1994; Felsenstein, 2004) and visualized with *FigTree* v.1.43 (Rambaut, 2012). We also used *MSA* to calculate pairwise values of *F*_ST_ values according to depth and among extant populations. We then used the *ape* package in R to conduct Mantel tests comparing pairwise values of linearized *F*_ST_ with depth or geographic distance, with estimates of significance based on 999 permutations. We undertook a Bayesian analysis implemented in the program *MIGRATE v3.6.11* to determine historical migration rates among sites within the Chesapeake (Beerli & Felsenstein, 1999) (Supporting Information Figure S2) with uniform priors and starting parameters set to Brownian motion for microsatellite data. We used F_ST_ calculations to determine theta and M values.

We also estimated genetic structure and genotypic variation using Bayesian approaches as implemented in *STRUCTURE* v.2.3.3 (Farrington & Petren, 2011; Pritchard, Stephens, & Donnelly, 2000). Separate analyses were carried out with data sets consisting of (a) depth cohorts; (b) depth cohorts and extant *S. americanus* in Kirkpatrick Marsh; (c) all samples from Chesapeake Bay; (d) all samples from the Atlantic coast; and (e) all samples from the Atlantic and Gulf coasts. A parallel series of analyses were completed with the full set of specimens for comparison to outcomes based on data sets excluding putative clones. For each *STRUCTURE* analysis, we allowed for admixture and correlated allele frequencies for three independent runs at iterative values of *K*, with the burn‐in period set to 30,000 iterations and data collected from an additional 500,000 iterations. Values of *K* were set to range from one to as high as 36 (i.e., across all sites where we sampled extant plants). The likeliest value of *K* was estimated according to the maximum Pr(*X*|*K*) value (Pritchard et al., 2000) and the break in the slope of the distribution of Pr(*X*|*K*) values (Evanno, Regnaut, & Goudet, 2005).

We visualized patterns of differentiation with genetic heat maps of optimal *K* estimates from *STRUCTURE* runs. Genetic cluster membership per individual served as the basis for interpolation using the Spatial Analyst Inverse Distance Weighted (IDW) Interpolation tool in *ArcGIS* (ESRI ArcMap v10.3). Inverse Distance Weighted utilizes a power function that assumes each sample site has a local influence that diminishes with increased distance; this function is used to weigh the points closer to the prediction location greater than those farther away. The result is a heat map of genetic relatedness between points based on cluster assignments and the distance between sites.

## 3 RESULTS

### 3.1 Seed bank profile, seed ages and seed germination

Seeds of *S. americanus* were recovered across the full length of the sediment cores taken in Kirkpatrick Marsh (Table 1, Figure 1). The maximum density of seeds from the 1997–2000 cores and core 2004‐A occurred between 18 cm and 24 cm (Table 1, Figure 1). The density of seeds declined precipitously at depths past 26 cm, although a spike in density was found at the 36–38 cm layer. ^210^Pb and ^137^Cs analysis of soil from core 2004‐A indicates that seeds recovered from layers above 30 cm correspond to a time period spanning 1875 (±92.8) to 2002 (±0.1).

We successfully germinated seeds that were recovered from depth layers dating from 1900 (±32.8) to 2002 (±0.1). At least one seed was recovered and germinated from soil layers spanning 0 to 24 cm depth intervals (Table 1). Seeds recovered from ≤24 cm depths germinated on average 6 days after planting (*SD* = 2.6) and no seeds germinated 14 days after planting. Germination rates differed according to seed age (*F*_3,10_ = 18.70, *p *= 0.0002). Post hoc comparisons of seeds recovered from the soil monolith indicate that seeds deposited in the 8–10 cm (1984 ± 1.2) depth had a significantly higher germination rate (52.2% ± 10.6 *SE*) compared to all other depth cohorts (range 3.3–13.3%; Table 1). However, germination rates were highest for seeds recovered from the 6–8 cm (1990 ± 1.3) and 10–12 cm (1984 ± 1.2) depths in core 2004‐A. When all sources were grouped, the highest germination rates occurred in cohorts recovered from 6–8 cm (1990 ± 1.3) and 10–12 cm (1976 ± 1.2) depths (Table 1). However, germination rates were statistically equivalent in soil depths above 14–16 cm (1947 ± 4.2), after which rates dropped by as much as 90% (Table 1). Germination rates were generally lower in our second assay than in our initial trial, particularly for seeds recovered from depths below 14 cm (Table 1).

### 3.2 Genetic diversity and effective population size through time

We examined 75 “resurrected” plants from six horizons spanning the 20th century: 2–4 cm (1998 ± 0.4), 8–10 cm (1984 ± 1.2), 12–14 cm (1963 ± 3.0), 14–16 cm (1947 ± 4.2), 20–22 cm (1908 ± 25), and 22–24 cm (1900 ± 32.2) (Table 2). To minimize potential artefacts due to small sample sizes, we grouped the single individual genotyped from the 1900 horizon with the individuals genotyped from the 1908 horizon, resulting in a single cohort spanning 1900–1908, and a total of five depth cohorts. An average of 15 individuals were genotyped per depth cohort, with the number of individuals per cohort varying between 5 and 40 individuals (Table 2). All “resurrected” individuals exhibited distinct genotypes.

No relationship was found between measures of genetic diversity and depth according to post hoc least‐squares linear regressions (all *r* ^*2*^ < 0.08, all *p* > 0.05). Genetic diversity across the length of the core could not be distinguished from a null, even distribution (*p* = 0.329). Similarly, *N*_e_ was not related to depth (*r* ^*2* = ^0.38, *p* = 0.16), nor did it deviate from an even distribution of *N*_e_ (*p* = 0.081) (Table 2). However, the 2–4 cm depth cohort exhibited a notably larger *N*_e_ than all the other depth cohorts (Table 2). Nonsignificant trends were recovered between estimates of genetic diversity and *N*_e_ with seed density (*r* = 0.63, *p* = 0.26; *r* = −0.40, *p* = 0.26, respectively).

### 3.3 Genetic and genotypic differentiation through time

**Figure 2:**
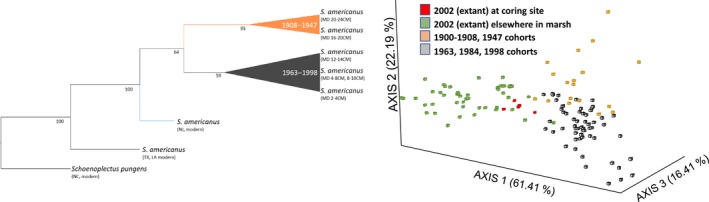
(Left) Neighbor‐joining tree of depth cohorts and select extant S. americanus (LA = Louisiana; MD = Maryland; NC = North Carolina; NJ = New Jersey; TX = Texas) and (Right) FCA of historical and extant genotypes from Kirkpatrick Marsh

**Figure 3:**
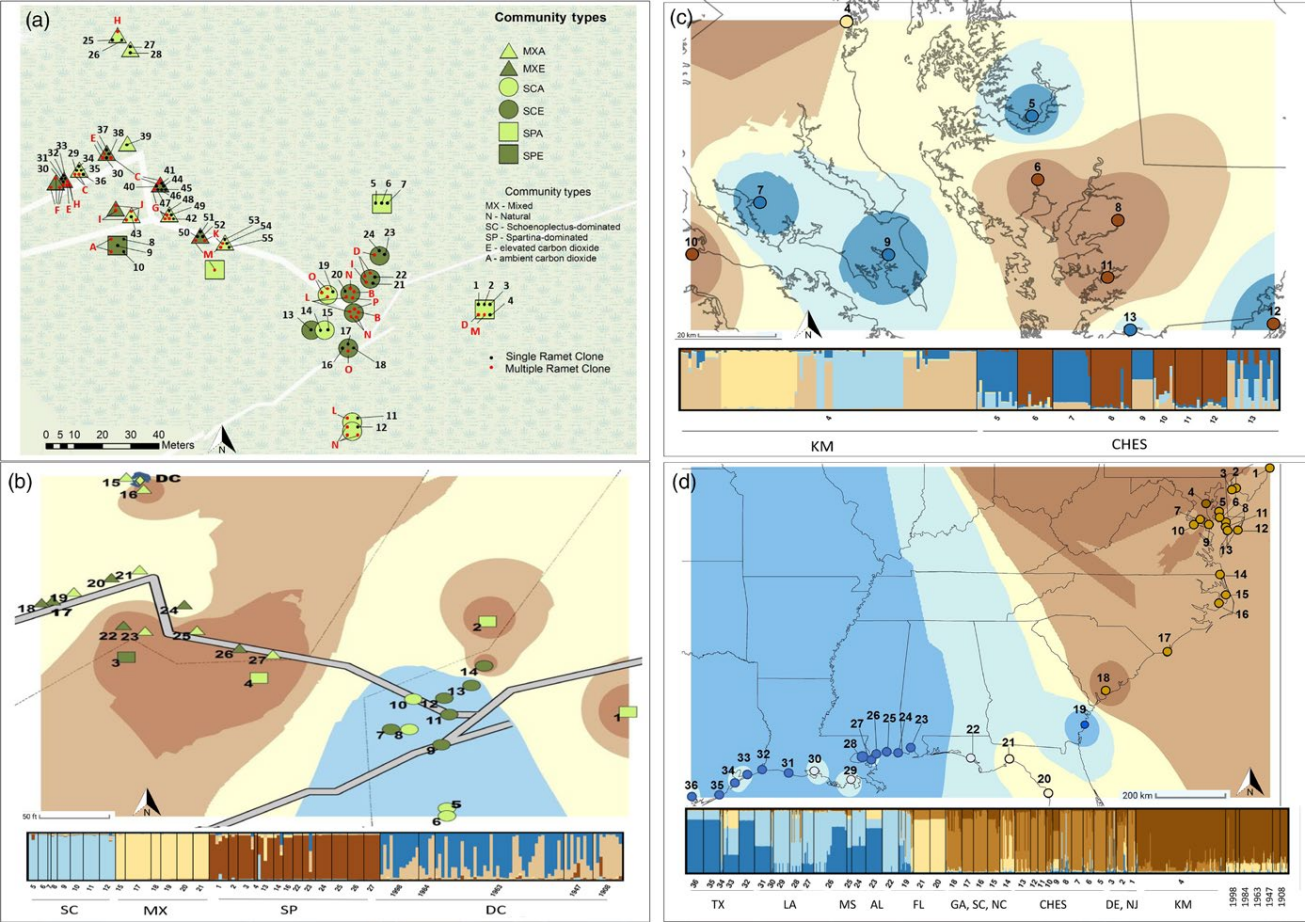
(a) Map of extant genotypic and clonal identity of *Schoenoplectus americanus* across Kirkpatrick Marsh. Genetic interpolation heat maps illustrating genetic relatedness based on optimal K from *STRUCTURE* of microsatellite allelic variation (b) within *S. americanus* depth cohorts from the Kirkpatrick marsh seed bank and extant samples from Kirkpatrick Marsh; (c) Chesapeake Bay marshes; (d) Atlantic and Gulf coast marshes. Shown for the best supported values of *K* as presented in the text. (a,b) Map of sample plots within Kirkpatrick Marsh

We detected evidence of genetic structure and genotypic shifts among depth cohorts. Approximately 3% of genetic variation was attributable to differences among depth cohorts, compared to 70% of variation attributable to differences within cohorts (Supporting Information Table S2). Mantel tests illustrated that genetic differentiation increased with increasing differences in depth (i.e., time) (Supporting Information Figure S1). *STRUCTURE* runs at *K* = 3 and *K* = 5 also showed that the genotypic composition of depth cohorts has shifted over time (Figure 3). Both the NJ dendrogram and FCA illustrated that a distinct shift between cohorts occurred across a depth horizon corresponding to *ca*. 1947 (Figure 2).

### 3.4 Comparison of historical and extant genetic variation in Kirkpatrick Marsh

With one exception (the *N*_e_ estimate for the 2–4 cm depth cohort), estimates of genetic diversity and *N*_e_ for individual depth cohorts were comparable to those estimated for extant *S. americanus* in Kirkpatrick Marsh and elsewhere (Table 2, Supporting Information Table S1). Combined estimates of genetic diversity and *N*_e_ for all cohorts were significantly higher than estimates for extant *S. americanus* in Kirkpatrick Marsh when all specimens were considered (Table 2). However, estimates were comparable between historical and extant *S. americanus* when putative clones were excluded from consideration (Table 2).

We detected evidence of genetic similarity among historical and extant *S. americanus*, as well as fine‐scale genetic structure among extant *S. americanus* across Kirkpatrick Marsh. The comparison of pairwise temporal distance and genetic distance between depth cohorts and extant individuals recovered a significant positive relationship, indicating that genetic differentiation between extant plants and cohorts progressively increases with time (Supporting Information Figure S1). The FCA of depth cohorts and extant *S. americanus* in Kirkpatrick Marsh (Figure 2) illustrates that extant plants from where the cores and monolith were recovered more closely resemble historical genotypes recovered from the three shallowest soil depths. *STRUCTURE* analyses further illustrate that extant plants in Kirkpatrick Marsh more closely resemble revived plants than extant plants from elsewhere in the Chesapeake (Figure 3). *STRUCTURE* analyses also show that variation in extant plants reflects fine‐scale differentiation corresponding to distance and community across Kirkpatrick Marsh (Figure 3). A Mantel test affirmed that genetic variation is associated with geographic distance across the marsh (Supporting Information Figure S1). An AMOVA showed that 23% of genetic variation is attributable to differences among plots when grouped by community (Supporting Information Table S2). The AMOVA of plots grouped by experimental treatment indicates that variance is not attributable to CO_2_ exposure regime (Supporting Information Table S2). Estimates of genetic diversity also did not differ according to exposure regime (all comparisons, *p* > 0.05), but mixed community plots exhibited significantly lower estimates of genetic diversity than both *Schoenoplectus*‐dominated plots (all comparisons; *p *≤ 0.05) and *Spartina*‐dominated plots (all comparisons *p *≤ 0.05). No differences were found between *Schoenoplectus*‐ and *Spartina*‐dominated plots.

Patterns of fine‐scale variation that appear to correspond to community type also parallel clone size and distribution across Kirkpatrick Marsh (Figure 3). Whereas all individuals from depth cohorts exhibited distinct genotypes, duplicate genotypes were detected at nearly every site where we sampled extant *S. americanus* (Table 2). Consequently, site‐level genotypic richness ranged from 0 to 1 (Table 2). In total, we detected duplicate genotypes in 333 samples (Table 2), including about half (55 of 109) of the specimens collected from Kirkpatrick Marsh plots (Table 2). This corresponded to a probability of <1.44e‐07 that shoots with identical genotypes were members of the same clone across the marsh, and a probability of <3.44e‐06 of sampling a second occurrence of each genotype given the number of genets sampled in the marsh. A clonal subrange value, which corresponds to the minimum estimate of the maximum distance between two identical genotypes (i.e., reflecting the distance for which the probability of clonal identity becomes zero), was estimated at approximately 43 m in Kirkpatrick Marsh (Alberto et al., 2005; Harada et al., 1997).

### 3.5 Genetic variation in extant *Schoenoplectus americanus*

Genetic diversity of depth cohorts and of extant *S. americanus* (excluding duplicate genotypes) in Kirkpatrick Marsh was comparable to levels of genetic diversity found in other Chesapeake Bay marshes (all comparisons *p* > 0.05). Estimates of genetic diversity also did not differ among Atlantic and Gulf coast sites (all comparisons *p* > 0.05), and no clear geographic patterns in diversity were observed across either coastline (Supporting Information Table S1).

We detected evidence of genetic structure across Chesapeake Bay marshes and across coastlines (Figure 3). Excluding duplicate genotypes, pairwise values of *F*_ST_ ranged from 0.06 to 0.48 among sample sites in Chesapeake Bay. An AMOVA attributed 27% (*p *< 0.001) of genetic variance to differences among sample sites. A Mantel test indicated that genetic distance corresponds to geographic distance (Supporting Information Figure S1); however, *STRUCTURE* runs at optimal *K* values (*K* = 4) revealed a more complex configuration of spatial differentiation in the embayment (Figure 3). Clusters aggregated nearby sites with one or two disjunct locations (Figure 3). Estimates of *N*_m_ between clusters ranged from 0.30 to 4.56 (Supporting Information Figure S2). We also detected a significant relationship between genetic distance and geographic distance across coastlines (Supporting Information Figure S1), and an AMOVA of all sites grouped by coast recovered a significant global *F*_ST_ of 0.23 (*p* < 0.001, Supporting Information Table S2). *STRUCTURE* runs with all unique genotypes (i.e., historical and extant samples) recovered clusters reflecting biogeographic breaks (i.e., Atlantic versus Gulf coast sites), as well as regional differences along coastlines (e.g., south Atlantic versus mid‐Atlantic sites). *STRUCTURE* runs with all unique genotypes also demonstrated that depth cohorts bear the greatest resemblance to extant *S. americanus* in Kirkpatrick Marsh (Figure 3).

## 4 DISCUSSION

Here we illustrate that, like other dormant propagule pools, soil‐stored seed banks can serve as a resource for studying demographic and genetic variation over time. Although concerns about biased representation and stratification have discouraged interest in soil‐stored seed banks as natural archives, our findings indicate that both can be constrained and potentially overcome. For example, concerns about biased representation can be minimized by examining species, like *S. americanus*, that exhibit prolific seed production and that produce highly persistent seeds that readily incorporate into the seed bank. Similarly, concerns about stratification can be overcome by examining seed banks that develop through sedimentary deposition. Although stringent, we have illustrated that it is possible to meet these conditions under relatively ordinary circumstances (i.e., by examining a widespread species found in a common environment). We have shown that *S. americanus* seeds can be recovered from radionuclide‐dated sedimentary layers spanning 100+ years. Comparisons of genetic diversity among contemporary populations and depth cohorts constructed from recovered seeds also indicate that postburial attrition and potential germination bias exert little influence on genetic measures of local demography. Evidence of shifting abundance, alongside spatial and temporal patterns of differentiation, further illustrate that soil‐stored seed banks can lend insight into the tempo and nature of ecological and evolutionary processes that shape populations over time.

Sedimentary records of coastal marshes, which have proven to be an exceptional resource for paleoecological reconstruction, exhibit features that facilitate use of soil‐stored seed banks as natural archives. Like sediments found in lakes (Hairston & Kearns, 2002) and coastal fjords (Härnström et al., 2011; Lundholm, Ribeiro, Godhe, Rostgaard Nielsen, & Ellegaard, 2017; Ribeiro, Berge, Lundholm, & Ellegaard, 2013), brackish marsh sediments are characteristically time‐stratified as a result of recurring deposition and accumulation (Kirwan & Murray, 2007). Bioturbation from animals like muskrats can be disruptive, but bioturbation is often highly localized; thus, the stratigraphic structure of marsh sediments typically remains well‐preserved (Kirwan & Murray, 2007; Stevenson & Hope, 2005). Sediment deposition and accumulation in marshes also can result in recurring burial and storage of seeds, particularly of seeds with durable coats (Fox, 1983; Honda, 2008; Moody‐Weis & Alexander, 2007) like those produced by *Schoenoplectus* sedges. In addition, other buried contents (e.g., diatoms) and attributes (e.g., mineral versus organic content, isotopic profiles) of marsh sediments can be examined to obtain information about past environmental conditions (e.g., inundation, salinity regimes) that determine plant performance (Kirwan & Murray, 2007; Park, Yu, Lim, & Shin, 2012). This can afford opportunities to relate proxy measures of plant demography like seed abundance with measures of environmental change over time (e.g., Jarrell et al., 2016; Saunders, 2003).

This study explores the prospects of exploiting a virtually untapped dimension of soil‐stored seed banks. Prior studies have largely utilized soil‐stored seed banks as resources to reconstruct records of past geological, climate‐related environmental conditions (e.g., Jarrell et al., 2016; Törnqvist et al., 2004). There is also an extensive literature on the contribution of seed banks to demography and genetic diversity (e.g., Cabin, Marshall, & Mitchell, 2000; Hegazy, Kabiel, Al‐Rowaily, Faisal, & Doma, 2014; Liu et al., 2014; Templeton & Levin, 1979). Little work has been done, however, on the use of soil‐stored seed banks for reconstructing records of genetic variation over time. Notably, McGraw, Vavrek, and Bennington (1991) highlighted the potential to do so by germinating *Carex bigelowii* and *Luzula parviflora* seeds recovered from tundra soil. Associated common garden experiments showed that depth cohorts of both species spanning ~150–200 years exhibited heritable differences in growth and morphological traits (Bennington et al., 1991; Vavrek et al., 1991). Using protein electrophoresis, Morris et al. (2002) also detected evidence of temporal variation among plants germinated from *Astragalus bibullatus* seeds recovered from successively deeper soil horizons sampled from the periphery of cedar glades in central Tennessee (USA). Our work further illustrates that genetic information can be extracted from soil‐stored seed banks and that it can be contextualized by a well‐constrained stratigraphic record as well as complementary information on local demography (i.e., shifts in seed densities) to draw inferences about ecological and evolutionary processes that shape populations over time.

We have shown that it is possible to overcome concerns about biased representation. As work on ephippia banks has demonstrated, a priori targeting a species with prolific seed production, like *S. americanus*, can reduce the likelihood of biased representation (Brendonck & De Meester, 2003; Cabin, 1996; Weider et al., 1997). Nonetheless, stochastic attrition and selection can bias the composition of dormant propagule banks over time (Weis, 2018). Biases can arise due to differences in germination at the time of seed production (Cabin, Mitchell, & Marshall, 1998; Levin, 1990; Mandák, Bímová, Mahelka, & Plačková, 2006) or if some seeds are more prone to decomposition or are less resilient to burial than others (Weis, 2018). Similarly, seed viability might vary, where some seeds are less likely to germinate after prolonged dormancy than others (Honda, 2008; Levin, 1990; Wagner & Oplinger, 2017; Weis, 2018). The *S. americanus* seed profile reconstructed from Kirkpatrick Marsh suggests that decomposition may have reduced seed abundance at depths greater than 40 cm, although it is possible that the decline in abundance instead reflects environmental conditions unfavorable to *S. americanus* (Jarrell et al., 2016). Thus, the observed decline may reflect historical trends in relative abundance and associated metrics like seed production rather than decomposition (Jarrell et al., 2016; Saunders, 2003). Germination rates, however, were only statistically equivalent for seeds recovered from depths up to 16 cm; rates dropped at greater depths (Table 1). While this suggests that burial is an important consideration, we did not detect genetic evidence that attrition or differences in germination biased the diversity of revived depth cohorts (Orsini et al., 2016). For example, we did not detect a loss of genetic diversity with increasing depth. This differs from prior studies that have detected aggregate measures of reduced genetic diversity (Cheliak, Dancik, Morgan, Yeh, & Strobeck, 1985; McCue & Holtsford, 1998; Orsini et al., 2016) and elevated genetic diversity in soil‐stored seed banks (Cabin, 1996; Mandák et al., 2006; Tonsor, Kalisz, Fisher, & Holtsford, 1993), which can arise due to selective differences in seed germination (Cabin, 1996; Levin, 1990; Mandák et al., 2006). Notably, we found that the genetic diversity of depth cohorts was comparable to the extant population, which is consistent with reports of genetic diversity in seed banks being a representative measure of local genetic variation (Honnay, Bossuyt, Jacquemyn, Shimono, & Uchiyama, 2008).

We also have demonstrated that it is possible to overcome concerns about stratigraphy. No signs of sediment mixing were evident in this study. Consistent with prior work in tundra and interior wetlands showing that dormant seeds can be recovered from age‐stratified soils (Bennington et al., 1991; McGraw et al., 1991; Vavrek et al., 1991), the laminate structure and radionuclide‐based age estimates of sediment sampled from Kirkpatrick Marsh demonstrated patterns of historical accumulation over a 150+ year period. A key next step, however, will be to reduce error rates of sediment age estimates. Error rates from ^210^Pb dating typically increase with depth (Table 1) (Binford, 1990; MacKenzie, Hardie, Farmer, Eades, & Pulford, 2011), and whereas ^137^Cs profiles can serve as referential benchmarks, more precise age estimates might be achieved through other approaches such as ^7^Be radionuclide dating (Olsen, Larsen, Lowry, Cutshall, & Nichols, 1986) or optically stimulated luminescence dating (Madsen, Murray, Andersen, Pejrup, & Breuning‐Madsen, 2005). Nevertheless, the observed pattern of progressive genetic differentiation over time (i.e., as opposed to genetic homogeneity) serves as supporting evidence that mixing did not disturb the sequence of the sampled stratigraphy (Orsini et al., 2016), as diversity and autocorrelation have been found to be lower in mixed sediment compared to undisturbed seed banks (England et al., 2003).

Local and range‐wide geographic comparisons offer an informative context for interpreting temporal patterns of genetic variation. We found that *S. americanus* exhibits a pattern of increasing dissimilarity with greater geographic distance, which is similar to patterns exhibited by other marsh plants (Blum, Jun Bando, Katz, & Strong, 2007; Mahy, Sloover, & Jacquemart, 1998; Travis & Hester, 2005; Travis, Proffitt, & Ritland, 2004). This, alongside evidence of genetic continuity and similarity between the seed bank and spatially proximate extant individuals in Kirkpatrick Marsh (Figure 2), indicates that immigration into the marsh is low (Supporting Information Figure S2) and that recruitment consistently draws from a local propagule pool (Honnay et al., 2008). Evidence that temporal variation is nested within spatial variation also indicates that genotypes “archived” in the soil‐stored seed bank are likely ancestral to genotypes in the extant population. Consistent with this, the observed patterns of hierarchically structured spatial genetic variation across the Chesapeake Bay suggest that individual or spatially proximate marsh complexes constitute (sub)populations connected by relatively low gene flow (Supporting Information Figures S1 and S2). Comparisons among marshes elsewhere on the Atlantic and Gulf coasts support this inference (results not shown), although we also detected genetic breaks corresponding to well‐recognized biogeographic discontinuities in North Atlantic coastal biota (Avise, 2000; Blum et al., 2007; Wares, 2002).

Our findings suggest that genetic variation in *S. americanus* reflects responses to biotic and abiotic conditions within marshes. Evidence of genetic continuity over time and low gene flow suggests that in situ (i.e., local) conditions likely exert a strong influence on genetic variation within marshes (Orsini et al., 2016). A number of factors are known to influence genetic variation in coastal marsh plants. Intrinsic organismal factors such as variation in asexual (i.e., vegetative tillering) and sexual reproduction can result in genetic mosaics like the one observed in Kirkpatrick Marsh, where diverse patches of small clones are juxtaposed with large swaths of individual clones (Hämälä, Mattila, Leinonen, Kuittinen, & Savolainen, 2017; Leck & Simpson, 1987; Richards, Hamrick, Donovan, & Mauricio, 2004). Estimates of N_e_ can similarly reflect the balance of asexual and sexual reproduction (López‐Villalobos & Eckert, 2018), as illustrated by the similar estimates of N_e_ recovered for all but one of the depth cohorts (Table 2), which are a product of sexual reproduction. Like other studies of marsh plants (Proffitt, Chiasson, Owens, Edwards, & Travis, 2005), we also found evidence suggesting that intraspecific and interspecific interactions (i.e., competition) play a role in structuring genetic variation in *S. americanus*. The observed pattern of differentiation in Kirkpatrick Marsh closely aligns with community type (i.e., *Schoenoplectus*‐dominated, *Spartina*‐dominated, or mixed), as do the size, number, and distribution of *S. americanus* clones (Emery, Ewanchuk, & Bertness, 2001; Erickson, Megonigal, Peresta, & Drake, 2007). It is possible, however, that this is a derivative outcome of microenvironmental shifts in stressors (e.g., salinity, inundation) that structure coastal marsh communities (Bertness & Ellison, 1987; Pennings & Callaway, 2000; Pennings, Grant, & Bertness, 2005).

Like the observed patterns of spatial variation, shifts in genotypic composition across depth cohorts might reflect responses to local selective pressures. Although it is possible that the observed pattern is a consequence of stochasticity (i.e., genetic drift), relatively modest changes in stressor exposure can structure whole marsh communities (Bertness & Ellison, 1987; Pennings et al., 2005), so by extension, shifts in stressor exposure might also structure genotypic composition within foundational marsh plants over time. Work on *Spartina alterniflora* supports this inference. For example, evidence has been found that stressor exposure (e.g., to oil, inundation) structures genetic variation across shoreline gradients (Anderson & Treshow, 1980; Gallagher, Somers, Grant, & Seliskar, 1988; Robertson, Schrey, Shayter, Moss, & Richards, 2017), although stressor responses may also reflect plasticity and epigenetic variation (Foust et al., 2016; Proffitt, Travis, & Edwards, 2003; Robertson et al., 2017). We incidentally assessed whether stressor exposure elicits genetic differentiation in *S. americanus* by drawing comparisons among FACE enclosures across Kirkpatrick Marsh. Prior work has shown that exposure to elevated CO_2_ increases *S. americanus* growth and reproduction (e.g., flowering), enough to shift the balance of competition in mixed communities toward *S. americanus* dominance (Arp et al., 1993; Langley & Megonigal, 2010; Rasse et al., 2005). Evidence also has been found for genotypic variation in responses of *S. americanus* to CO_2_ exposure (Gentile, 2015), and studies conducted at other FACE sites have shown that experimental exposure to CO_2_ can result in rapid adaptive responses in plants (Grossman & Rice, 2014). We did not find evidence, however, that genetic variation is associated with CO_2_ exposure across the GCReW site. A more thorough assessment (e.g., SNP‐based genomic analyses) might uncover signatures of responses to CO_2_ exposure, although it is also possible that responses to stressors that reduce fitness and elevate mortality (e.g., increasing salinity and inundation) might supersede signatures of response to CO_2_.

Addressing some of the methodological limitations that we encountered could help foster further development and use of soil‐stored seed banks as natural archives. Achieving larger sample sizes, for example, would offer a stronger basis for inferring patterns of genetic variation over time. As reconstituting depth cohorts is a process of diminishing returns, future work could improve upon our efforts by sampling a larger volume of soil (i.e., by taking more and/or larger sediment cores). This would help overcome limitations set by shifts in abundance over time (Jarrell et al., 2016) and low germination rates, particularly for reconstituting cohorts from deeper (i.e., >16 cm) soil layers. Reconstituting cohorts from finer scale depth intervals could also minimize discontinuities (i.e., time steps) and thus offer a stronger basis for examining dynamic demographic processes like population turnover (Ponnikas, Ollila, & Kvist, 2017). It may be possible to increase sample sizes by increasing germination rates, although trials so far conducted suggest that methodological modifications may only lead to marginal improvements (Gentile, 2015). Drawing comparisons across sites (i.e., by examining depth cohorts reconstituted from cores taken at multiple locations) would clarify whether the patterns observed in this study reflect general phenomena or conditions idiosyncratic to Kirkpatrick Marsh. Separately genotyping seed coats and germplasm would also be a key step toward understanding the limits of inferences that can be drawn from plants derived from buried seeds. This would not only clarify whether depth cohorts are representative of the seed bank, it would offer a basis for inferring relatedness and possibly a basis for reconstructing pedigrees (i.e., seed coats are typically maternally derived, whereas germplasm reflect biparental contributions).

Besides demonstrating that soil‐stored seed banks can offer perspectives on demographic and genetic change over time, our work illustrates that dormant soil‐stored seeds can be a resource for experimental “resurrection” approaches for studying ecological and evolutionary responses of plants to environmental change over time. In many ways, the process of reconstituting depth cohorts from soil‐stored seed banks parallels the steps required to assemble experimental cohorts from dormant zooplankton ephippia and curated seed collections (Franks & Weis, 2008; Franks et al., 2007). Thus, the literature on both can serve as guides for pursuing further work to improve use of soil‐stored seed banks as a resource for “resurrection” studies. For example, besides improvement of propagation and germination methods, conducting test crosses to develop pedigreed lines could help augment sample sizes and enable the analysis of trait heritability (e.g., Franks et al., 2007), including traits that contribute to seed survival and germination. And, as has been done with zooplankton hatched from dormant ephippia, elaborating on the genomic and transcriptomic variation in responses to stressor exposure could offer greater insight into the role of drift and selection in shaping temporal patterns of genetic variation (Orsini et al., 2016). Likewise, stronger inferences could be drawn by characterizing longer time horizons (e.g., Frisch et al., 2014) and drawing comparisons to independent records of environmental change. Doing so would not only increase confidence in the use of soil‐stored seed banks for the study of coastal marshes, it would also foster further interest in the use of soil‐stored seed banks (Bennington et al., 1991; McGraw et al., 1991; Morris et al., 2002; Vavrek et al., 1991) for examining other ecosystems (e.g., tundra, interior wetlands) that are highly vulnerable to climate change and land use intensification.

## DATA ARCHIVING

Data available from the Dryad Digital Repository: https://doi.org/10.5061/dryad.c76q3t7.
