# Supplementary material for: A century of genetic variation inferred from a persistent soil‐stored seed bank
Source: Evol Appl. 2018 Jul 29;11(9):1715–31. doi: 10.1111/eva.12675 (PMC6183470; doi:10.1111/eva.12675)
Supplement: Supplementary file 1 [file EVA-11-1715-s001.pdf]

Figure S1

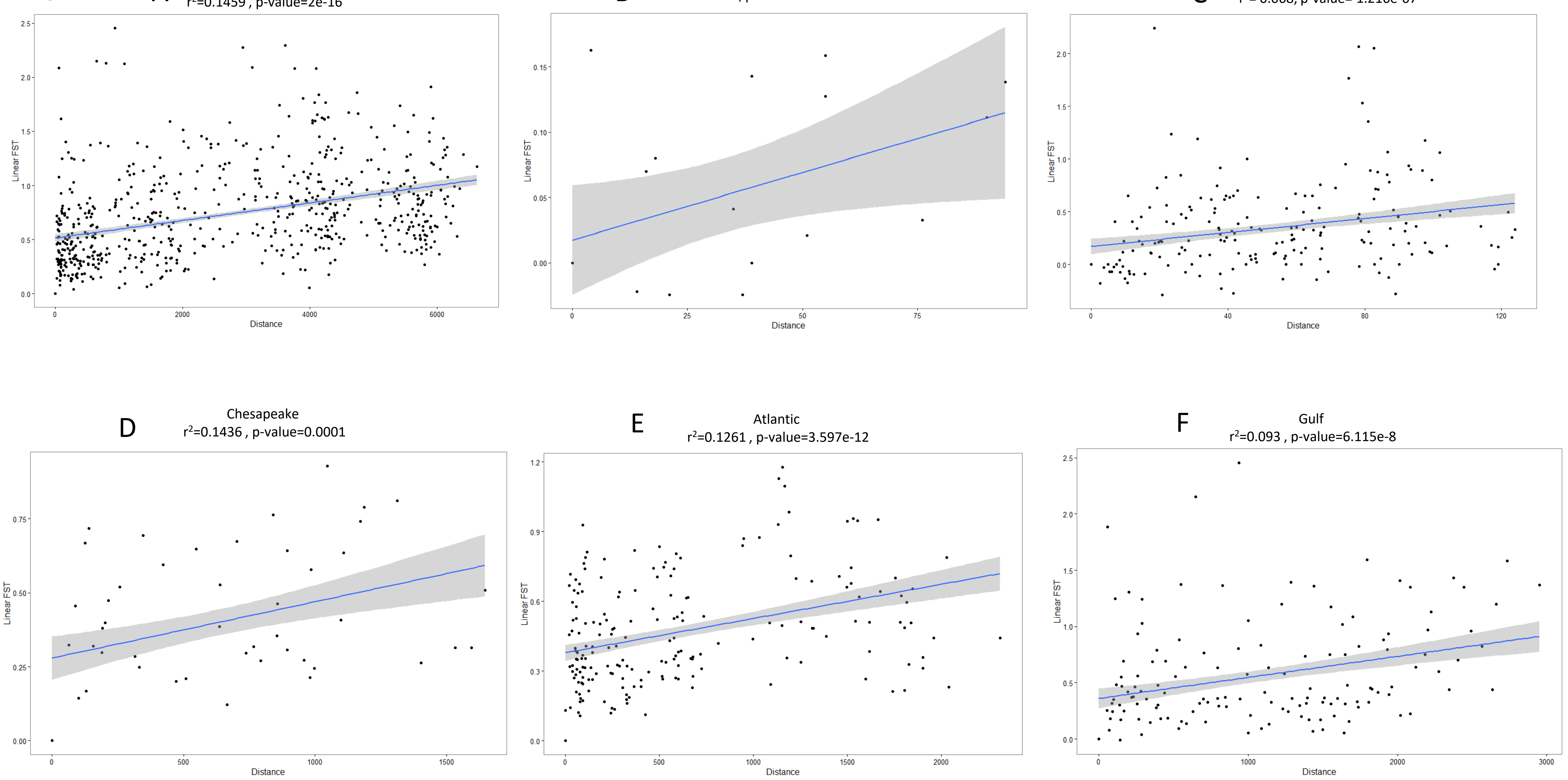
A
All
r^2=0.1459 , p-value=2e-16
B
Depth cohorts and extant Kirkpatrick
r^2=0.1789, p-value=0.03593
C
Kirkpatrick
r^2= 0.068, p-value= 1.216e-07
D
Chesapeake
r^2=0.1436 , p-value=0.0001
E
Atlantic
r^2=0.1261 , p-value=3.597e-12
F
Gulf
r^2=0.093 , p-value=6.115e-8
Linear FST
Distance

Figure S2

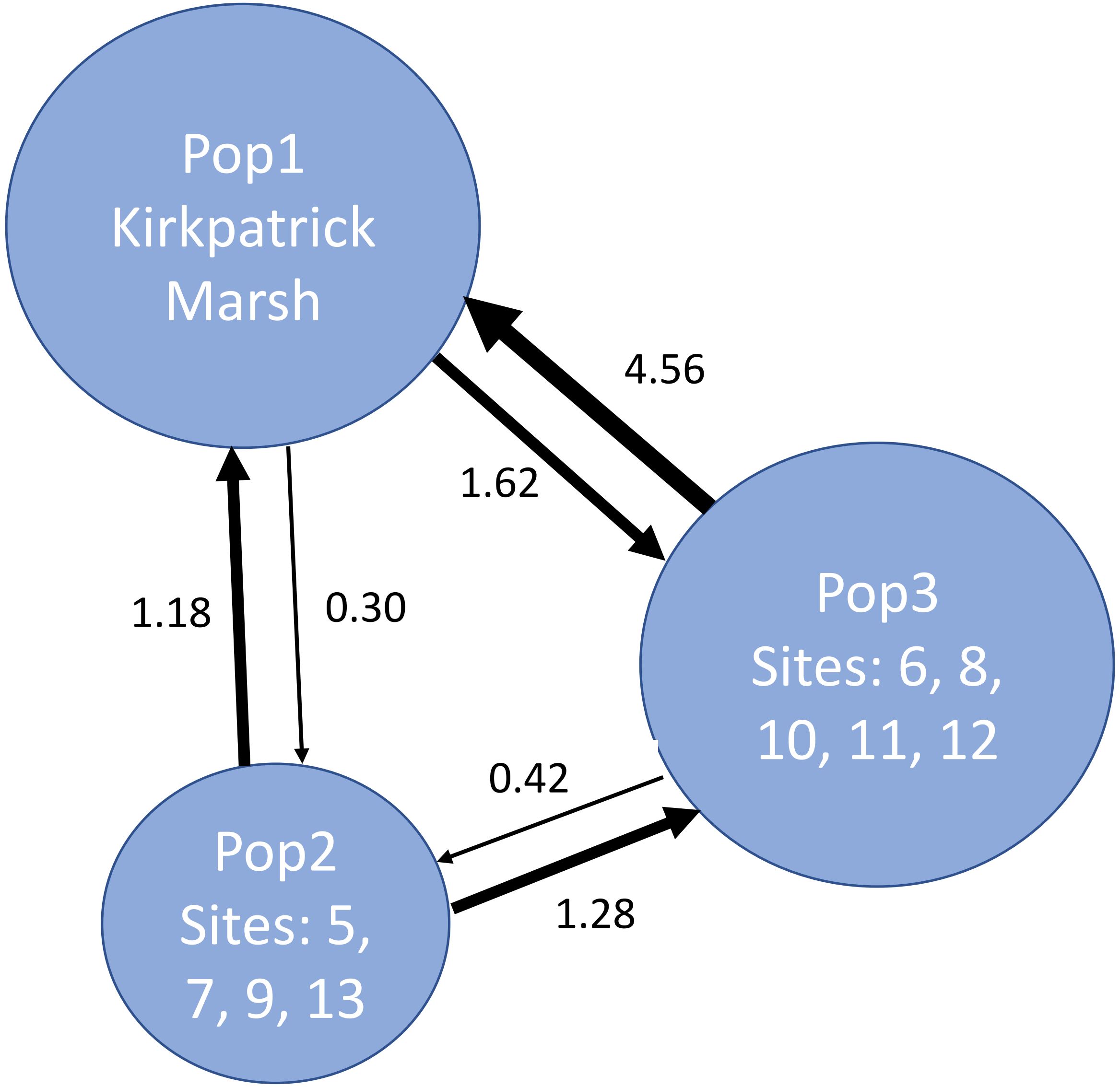
Pop1
Kirkpatrick
Marsh
Pop2
Sites: 5,
7, 9, 13
Pop3
Sites: 6, 8,
10, 11, 12
4.56
1.62
1.18
0.30
0.42
1.28
