# Supplementary material for: A century of genetic variation inferred from a persistent soil‐stored seed bank
Source: Evol Appl. 2018 Jul 29;11(9):1715–31. doi: 10.1111/eva.12675 (PMC6183470; doi:10.1111/eva.12675)
Supplement: Supplementary file 2 [file EVA-11-1715-s002.docx]

Table S1: Genetic variation of extant *S. americanus* from additional Chesapeake Bay marshes and marshes across the Atlantic and Gulf coasts of North America.

| Site # | | Location | State | Latitude (N) | Longitude(W) | N | G | Ne | Clones Excluded | | |  | Clones Included | | |
| --- | --- | --- | --- | --- | --- | --- | --- | --- | --- | --- | --- | --- | --- | --- | --- |
|  | |  |  |  |  |  |  |  | R | He | S |  | He | S | Ar |
| Chesapeake Bay marshes | | |  |  |  |  |  |  |  |  |  |  |  |  |  |
|  |  | Kirkpatrick marsh depth cohorts | MD | 38°52'26.63" | 76°32'53.16" | 75 | 75 | 38.5 | 1.00 | 0.44 | 0.87 |  | 0.44 | 0.87 | 1.44 |
| 4 |  | Kirkpatrick marsh (W) | MD | 38°52'26.63" | 76°32'53.16" | 109 | 54 | 7.5 | 0.50 | 0.26 | 0.55 |  | 0.48 | 0.87 | 1.48 |
| 5 |  | Choptank River (E) | MD | 38°36'0.00" | 76° 0'60.00" | 15 | 12 | 0.9 | 0.80 | 0.29 | 0.47 |  | 0.34 | 0.54 | 1.34 |
| 6 |  | Fishing Bay (E) | MD | 38°24'60.00" | 76° 0'0.00" | 13 | 13 | 57.6 | 1.00 | 0.51 | 0.89 |  | 0.51 | 0.89 | 1.51 |
| 7 |  | Chaptico Run (W) | MD | 38°21'0.00" | 76°47'60.00" | 14 | 6 | 1.7 | 0.43 | 0.19 | 0.29 |  | 0.31 | 0.45 | 1.31 |
| 8 |  | Wicomico River (E) | MD | 38°17'60.00" | 75°45'60.00" | 15 | 15 | 16.2 | 1.00 | 0.45 | 0.76 |  | 0.45 | 0.76 | 1.45 |
| 9 |  | St Mary's River (W) | MD | 38°12'0.00" | 76°26'0.00" | 8 | 2 | 0.7 | 0.25 | 0.31 | 0.42 |  | 0.38 | 0.41 | 1.38 |
| 10 |  | Rosier Creek (W) | VA | 38°12'0.00" | 77° 0'0.00" | 8 | 7 | 1.4 | 0.88 | 0.26 | 0.45 |  | 0.33 | 0.53 | 1.33 |
| 11 |  | Manokin River (E) | MD | 38°07'60.00" | 75°47'60.00" | 10 | 10 | 12.5 | 1.00 | 0.37 | 0.60 |  | 0.37 | 0.60 | 1.37 |
| 12 |  | Purnell Pond (E) | MD | 37°060'0.00" | 75°18'60.00" | 9 | 8 | 4.1 | 0.89 | 0.32 | 0.56 |  | 0.47 | 0.73 | 1.47 |
| 13 |  | Richardson Marsh (E) | MD | 37°59'0.00" | 75°44'0.00" | 19 | 7 | 0.9 | 0.37 | 0.26 | 0.41 |  | 0.28 | 0.41 | 1.28 |
|  |  |  |  |  |  |  |  |  |  |  |  |  |  |  |  |
|  |  |  |  |  | total | 111 | 80 |  | 0.74 | 0.33 | 0.57 |  | 0.40 | 0.64 | 1.40 |
| Other Atlantic coast marshes | | |  |  |  |  |  |  |  |  |  |  |  |  |  |
| 1 |  | Metedeconk Neck | NJ | 40° 1'60.00" | 74° 3'60.00" | 10 | 9 |  | 0.90 | 0.29 | 0.41 |  | 0.36 | 0.53 | 1.36 |
| 2 |  | Delaware Bay | NJ | 39°22'60.00" | 75°24'0.00" | 14 | 14 |  | 1.00 | 0.45 | 0.78 |  | 0.45 | 0.78 | 1.45 |
| 3 |  | Delaware River | DE | 39°21'0.00" | 75°32'60.00" | 12 | 9 |  | 0.75 | 0.29 | 0.47 |  | 0.36 | 0.57 | 1.36 |
| 14 |  | Mackay Island | NC | 36°31'60.00" | 75°59'0.00" | 18 | 8 |  | 0.44 | 0.34 | 0.60 |  | 0.38 | 0.63 | 1.38 |
| 15 |  | Croatan Sound | NC | 35°52'60.00" | 75°45'60.00" | 15 | 9 |  | 0.60 | 0.26 | 0.38 |  | 0.31 | 0.44 | 1.31 |
| 16 |  | Pungo River | NC | 35°34'0.00" | 76° 2'60.00" | 17 | 15 |  | 0.88 | 0.29 | 0.46 |  | 0.30 | 0.48 | 1.30 |
| 17 |  | Oak Island | NC | 33°54'60.00" | 78° 3'60.00" | 14 | 6 |  | 0.43 | 0.22 | 0.34 |  | 0.27 | 0.39 | 1.27 |
| 18 |  | Bear Island WMA | SC | 32°35'18.84" | 80°27'42.06" | 20 | 8 |  | 0.40 | 0.35 | 0.54 |  | 0.43 | 0.63 | 1.46 |
| 19 |  | Sapelo Island | GA | 31°23'50.00" | 81°16'43.08" | 18 | 16 |  | 0.89 | 0.41 | 0.73 |  | 0.42 | 0.75 | 1.53 |
|  |  |  |  |  |  |  |  |  |  |  |  |  |  |  |  |
|  |  |  |  |  | total | 138 | 94 |  | 0.70 | 0.32 | 0.52 |  | 0.36 | 0.58 | 1.38 |
| Gulf coast marshes | | |  |  |  |  |  |  |  |  |  |  |  |  |  |
| 20 | Crystal River | | FL | 28°54'39.42" | 82°41'32.28" | 19 | 3 |  | 0.16 | 0.19 | 0.26 |  | 0.35 | 0.44 | 1.35 |
| 21 | St Mark's River | | FL | 30° 9'15.90" | 84°12'16.98" | 20 | 3 |  | 0.15 | 0.20 | 0.32 |  | 0.34 | 0.43 | 1.34 |
| 22 | St. Andrew Bay | | FL | 30°11'18.48" | 85°43'31.26" | 20 | 2 |  | 0.10 | 0.30 | 0.42 |  | 0.39 | 0.44 | 1.39 |
| 23 | Mobile Bay | | AL | 30°34'15.72" | 88° 5'10.92" | 20 | 3 |  | 0.15 | 0.34 | 0.49 |  | 0.45 | 0.58 | 1.45 |
| 24 | Pascagoula Bay | | MS | 30°23'35.46" | 88°32'53.70" | 17 | 15 |  | 0.88 | 0.16 | 0.25 |  | 0.20 | 0.31 | 1.20 |
| 25 | Biloxi Bay | | MS | 30°25'60.00" | 88°59'0.00" | 6 | 1 |  | 0.17 | 0.32 | 0.45 |  | 0.64 | 0.44 | 1.64 |
| 26 | Bay St. Louis | | MS | 30°19'44.40'' | 89°25'28.92" | 40 | 16 |  | 0.40 | 0.42 | 0.68 |  | 0.48 | 0.75 | 1.48 |
| 27 | Pearl River | | LA | 30° 11'0.00" | 89° 35'00.00" | 3 | 3 |  | 1.00 | 0.59 | 0.80 |  | 0.59 | 0.80 | 1.41 |
| 28 | Lake Pontchartrain, Big Branch Marsh | | LA | 30°15'55.08'' | 89°57'58.80'' | 14 | 12 |  | 0.86 | 0.34 | 0.62 |  | 0.41 | 0.69 | 1.42 |
| 29 | Point Aux Chenes WMA | | LA | 29°25'29.94" | 90°23'40.62" | 15 | 7 |  | 0.47 | 0.38 | 0.64 |  | 0.42 | 0.67 | 1.44 |
| 30 | West Cote Blanche Bay | | LA | 29°44'37.50'' | 91°48'60.00'' | 20 | 2 |  | 0.10 | 0.37 | 0.58 |  | 0.44 | 0.53 | 1.59 |
| 31 | Rockefeller State Wildlife Refuge | | LA | 29°41'10.96" | 92°50'31.46" | 20 | 9 |  | 0.45 | 0.22 | 0.36 |  | 0.31 | 0.45 | 1.31 |
| 32 | Sabine Lake | | LA | 29°46'18.48'' | 93°51'5.40'' | 19 | 2 |  | 0.11 | 0.38 | 0.55 |  | 0.47 | 0.53 | 1.47 |
|  |  | |  |  |  |  |  |  |  |  |  |  |  |  |  |
| 33 | Galveston Bay, Anahuac NWR | | TX | 29°37'1.50" | 94°26'15.84" | 19 | 4 |  | 0.21 | 0.38 | 0.59 |  | 0.49 | 0.72 | 1.49 |
| 34 | Galveston Bay, Bayou Vista | | TX | 29°18'18.05 | 94°53'58.02" | 4 | 4 |  | 1.00 | 0.62 | 0.92 |  | 0.62 | 0.92 | 1.62 |
| 35 | San Bernard NWR | | TX | 28°52'51.24" | 95°33'36.42" | 20 | 2 |  | 0.10 | 0.21 | 0.28 |  | 0.23 | 0.24 | 1.23 |
| 36 | Lavaca Bay | | TX | 28°48'19.32" | 96°36'46.86" | 20 | 5 |  | 0.25 | 0.16 | 0.27 |  | 0.30 | 0.45 | 1.30 |
|  |  | |  |  |  |  |  |  |  |  |  |  |  |  |  |
|  |  | |  |  | total | 296 | 93 |  | 0.39 | 0.33 | 0.50 |  | 0.42 | 0.55 | 1.42 |
|  |  | |  |  | Overall total | 545 | 267 |  |  |  |  |  |  |  |  |

Sample site numbers correspond to those given in Figure 3. Sample size is given as (*N*); *G* = number of unique multi-locus genotypes; *R* = genotypic richness; *H_e_* = expected heterozygosity; *S* = Shannon Diversity Index values; and AR = rarified allelic richness; N_e_ = effective population size. Diversity measures were calculated with and without putative clones. Putative clones were excluded from N_e_ calculations.

Table S2: Analysis of molecular variance (AMOVA) in *Schoenoplectus americanus* according to time, geography, community type, and across CO_2_ exposure regimes

| Source of variation | DF | SS | % of total variation | PhiPT | Nm | p-value |
| --- | --- | --- | --- | --- | --- | --- |
| 1. Among all depth cohorts   Within cohorts  Total | 4  70  74 | 50.189  447.664  497.853 | 7%  93%  100% | 0.072 | 3.200 | 0.003 |
| (b) Among depth cohorts and extant *S. americanus* in Kirkpatrick Marsh (KM)  Within populations  Total | 1  127  128 | 90.287  885.798  976.085 | 16%  84%  100% | 0.160 | 1.314 | 0.001 |
| (c) Among CO_2_ exposure regime across KM  Among plots  Within plots  Total | 1  15  37  53 | 14.815  204.013  169.117  387.944 | 0%  39%  61%  100% | 0.387 | 0.396 | 0.001 |
| (d) Among community plot types in KM  Among plots  Within plots  Total | 2  14  37  53 | 84.856  133.971  169.117  387.944 | 23%  20%  56%  100% | 0.435 | 0.325 | 0.001 |
| (e) Among extant KM & all other Chesapeake Bay (CM) marshes  Among all CM  Within all CM  Total | 1  132  133  266 | 73.929  315.840  743.142  1132.910 | 0%  39%  61%  100% | 0.313 | 0.549 | 0.001 |
| (f) Among extant Atlantic and Gulf marshes  Among all marshes  Within all marshes  Total | 1  33  286  320 | 353.079  1279.001  1576.353  3208.433 | 20%  33%  48%  100% | 0.523 | 0.228 | 0.001 |
